# The rocky road to personalized medicine in acute myeloid leukaemia

**DOI:** 10.1111/jcmm.13478

**Published:** 2018-01-12

**Authors:** Bryan Brinda, Irum Khan, Brian Parkin, Heiko Konig

**Affiliations:** ^1^ Indiana University Melvin and Bren Simon Cancer Center Indianapolis IN USA; ^2^ Division of Hematology and Oncology College of Medicine at Chicago University of Illinois Chicago IL USA; ^3^ University of Michigan Comprehensive Cancer Center Ann Arbor MI USA

**Keywords:** acute myeloid leukaemia, targeted therapies, drug resistance, minimal residual disease

## Abstract

Acute myeloid leukaemia (AML) is a malignant disorder of the myeloid blood lineage characterized by impaired differentiation and increased proliferation of hematopoietic precursor cells. Recent technological advances have led to an improved understanding of AML biology but also uncovered the enormous cytogenetic and molecular heterogeneity of the disease. Despite this heterogeneity, AML is mostly managed by a ‘one‐size‐fits‐all’ approach consisting of intensive, highly toxic induction and consolidation chemotherapy. These treatment protocols have remained largely unchanged for the past several decades and only lead to a cure in approximately 30–35% of cases. The advent of targeted therapies in chronic myeloid leukaemia and other malignancies has sparked hope to improve patient outcome in AML. However, the implementation of targeted agents in AML therapy has been unexpectedly cumbersome and remains a difficult task due to a variety of disease‐ and patient‐specific factors. In this review, we describe current standard and investigational therapeutic strategies with a focus on targeted agents and highlight potential tools that might facilitate the development of targeted therapies for this fatal disease. The classes of agents described in this review include constitutively activated signalling pathway inhibitors, surface receptor targets, epigenetic modifiers, drugs targeting the interaction of the hematopoietic progenitor cell with the stroma and drugs that target the apoptotic machinery. The clinical context and outcome with these agents will be examined to gain insight about their optimal utilization.

**Table:** 

• **Introduction**
• **Current approaches to management of AML**
• **Heading towards targeted therapies for AML**
‐ Surface receptors
‐ KIT
‐ FLT3
‐ RAS
‐ Polo‐like kinases
‐ Cyclin‐dependent kinase inhibitors
‐ Targeting apoptosis
‐ Targeting the stroma
‐ Epigenetics
‐ DNMT inhibitors
‐ IDH inhibitors
‐ HDAC inhibitors
‐ DOT1L inhibitors
‐ Bromodomain inhibitors
• **AML heterogeneity and minimal residual disease**
• **Conclusion**
• **Conflict of interest**

## Introduction

Personalized cancer therapy offers the hope to establish novel and more effective therapeutic standards for patients afflicted with this condition. While traditional chemotherapeutic protocols aim to destroy rapidly dividing cells, but also affect normal (‘healthy’) cells, personalized medicine represents a promising concept by which patients whose cancer cells harbour pathophysiologically and therapeutically relevant molecular alterations could be treated with a biomarker‐based ‘targeted’ therapy. In the long term, this strategy may be cost‐effective, even including the required diagnostic and follow‐up tests that accompany therapy (so‐called companion diagnostics). Personalized medicine has become a synonym for the medicine of the future to which many experts ascribe a paradigm change. The overwhelming success of the tyrosine kinase inhibitor imatinib 1 and the monoclonal, CD20‐targeted antibody rituximab 2 has revolutionized the care of patients with chronic myeloid leukaemia (CML) and non‐Hodgkin lymphoma, respectively, and validated the use of targeted treatment strategies in the management of patients with cancer. Acute myeloid leukaemia (AML) is an aggressive form of cancer of the bone marrow (BM) and blood that is characterized by blocked differentiation and rapid proliferation of myeloid precursor cells. Despite major advances in understanding AML at the molecular level, novel treatment concepts are lacking 3. Therapeutic concepts to manage AML have remained largely unchanged since the 1970s and frequently fail to achieve a cure, underscored by 5‐year survival rates of roughly only 30% 4. The current concept of the molecular basis of AML suggests that the disease arises in hematopoietic precursor cells and is driven by at least two types of cooperative mutations (‘the two hit model’). However, novel technologies such as genome sequencing have unveiled a much more complex picture of leukemogenesis and shed further light on hitherto unknown obstacles in the way to targeted therapy for AML.

## Current approaches to management of AML

Current standard treatments for AML consist of induction chemotherapy followed by several courses of consolidation chemotherapy or allogeneic stem cell transplantation (aSCT). Herein, induction protocols mostly employ the so‐called 7 + 3 regimen, which entails continuous infusion cytarabine given over 7 days and 3 days of an anthracycline, typically either daunorubicin or idarubicin. Although the ideal dose of daunorubicin remains an open question, this approach has remained unchanged for the past several decades 5, 6, 7. The combination of cytarabine and an anthracycline as intensive remission therapy produces complete remission (CR) rates of 60–80% and 40–60% in patients that are less than age 60 and age 60 or greater, respectively 8. The lower CR rate in elderly patients is a reflection of decreased sensitivity of leukaemic cells to chemotherapy as well as a decreased tolerance to therapy and increased treatment‐related mortality 9. However, even in younger patients, standard AML induction and consolidation regimens frequently lead to complications, such as cytopenias and infections as well as gastroenteric and neurologic toxicities. In addition, only a minority of patients are cured by this approach which highlights the urgent need for novel and improved treatment concepts. Of note, CPX‐351, a liposomal combination of daunorubicin and cytarabine, was recently approved by the FDA for intensive remission induction in adults with newly diagnosed therapy‐related AML or AML with myelodysplasia‐related changes. The approval was based on the results of a phase III clinical trial where CPX‐351 significantly improved overall survival, event‐free survival and response without an increase in 60‐day mortality compared to standard ‘7 + 3’ chemotherapy 10.

## Heading towards targeted therapies for AML

### Surface receptors

Gemtuzumab ozogamicin (GO), an anti‐CD33 immunoconjugate, has the unique distinction of being the first targeted agent in AML that was approved by the FDA *via* accelerated approval in 2000 for older patients with AML in first relapse 11. The drug was subsequently withdrawn from the U.S. market in June 2010 after a randomized study by SWOG failed to demonstrate improved efficacy, while induction mortality was increased compared to the chemotherapy alone arm 12. To refute these findings, four subsequent randomized studies 13, 14, 15, 16 strongly support the safety and efficacy of this agent in combination with upfront chemotherapy in AML. The addition of GO significantly reduced relapse and improved overall survival at 5 years, with this benefit being most prominent in patients with favourable or intermediate‐risk cytogenetics 17. The inferior outcomes of the SWOG study were attributed to lower anthracycline dosing in the GO arm as well higher doses of GO causing veno‐occlusive disease (VOD). GO has also been combined with the hypomethylating agents (HMAs) 18, 19 based on the observation that azacitidine induces CD33 expression and decreases *P*‐glycoprotein expression, with favourable response rates of 35–44%. Unfortunately, a randomized study where GO was added to low‐dose cytarabine did not translate into improved survival 20. Building on the lessons gained from GO, vadastuximab talirine (SGN‐33A), another CD33‐directed, antibody–drug conjugate that employs pyrrolobenzodiazepine instead of calicheamicin, was developed. A phase I study of vadastuximab in combination with an HMA (azacitidine or decitabine) 21 in untreated patients unfit for intensive therapy reported complete remission and complete remission with incomplete count recovery (CR/CRi) rates of 73% among evaluable patients. In combination with induction chemotherapy, vadastuximab produced a CR/CRi rate of 78%, with 30‐ and 60‐day mortality of 0 and 7%, respectively 22. While these preliminary findings are encouraging, additional studies are currently ongoing to further evaluate the role of vadastuximab in AML therapy (Table 1).

**Table 1:** Targeted agents under clinical investigation either a single agent or in combination with acute myeloid leukaemia therapy

Target category	Drug target	Drug	Trial phase	Patient population [Results]	Single agent/combination	Ref./identifier	Status
Cell surface receptors	CD33	Gemtuzumab ozogamicin	III	3325 adult patients with the first course of intensive remission chemotherapy.	Combination with induction chemotherapy	16	Completed
			II	Patients age 60 and greater with newly diagnosed AML.	Combination with azacitidine	17	Completed
		Vadastuximab talirine	I/II	Pre‐allogeneic transplant (with conditioning regimen) OR post‐allogeneic transplant (single agent) in adults >18 years	Single agent and combination	NCT02614560	Active, not recruiting
			III	Adult patients with newly diagnosed AML	Combination with azacitidine OR decitabine	NCT02785900	Recruiting
			I	Safety study as a single agent and in combination with HMA to determine the maximum tolerated dose in adult patients >18 years	Single agent and combination with HMA	NCT01902329	Active, not recruiting
Tyrosine kinase pathways	c‐kit	Dasatinib	I	Children and adolescent patients with CBF AML to determine maximum tolerated dose	Combination with induction therapy	NCT02680951	Recruiting
			Ib/IIa	Given after induction and consolidation for maintenance therapy for 1 year in adult patients >18 years	Single agent (maintenance)	NCT00850382	Completed, results not available
			II	Given after consolidation for patients with high‐risk MRD or in molecular relapse in adults age 18–60 years	Single agent (maintenance)	NCT02113319	Completed, results not available
			III	Standard induction and consolidation therapy with or without dasatinib in adults age >18 years	Combination	NCT02013648	Recruiting
	FLT3	Midostaurin	III	Adult patients up to age 60 with newly diagnosed FLT3‐mutated AML. CR 59% *versus* 54%, OS 74.7 *versus* 25.6 months	Combination with induction, consolidation, and maintenance *versus* placebo	NCT00651261 32, 115	Completed^a^
			I	Adults age 60 and greater with newly diagnosed AML or relapsed/refractory disease	Combination with decitabine	NCT01130662	Completed, results not available
			I	Adults with newly diagnosed AML	Combination with daunorubicin and cytarabine induction	NCT00093600	Completed, results not available
			I	Adults with relapsed/refractory AML	Combination with bortezomib and cytotoxic chemotherapy	NCT01174888	Completed, results not available
			I/II	Adult patients with relapsed/refractory AML or newly diagnosed AML who are ineligible to receive intensive therapy	Combination with azacitidine	NCT01093573	Active, not recruiting
			II	Patients with AML having received allogeneic HSCT	Single agent (maintenance)	NCT02723435	Not yet open
			II/III	Patients age 60 or older with previously untreated AML	Combination with azacitidine and nivolumab	NCT03092674	Not yet open
		Sorafenib	I	Patients age 60 or older with relapsed/refractory or newly diagnosed AML who are not eligible to receive intensive therapy	Combination with bortezomib and decitabine	NCT01861314	Active, not recruiting
			IV	Patients status post‐allogeneic HSCT with FLT3/ITD mutation in adults age 18‐60 years	Single agent (maintenance)	NCT02474290	Recruiting
			II	Adult patients less than 60 years old with newly diagnosed AML; event‐free survival 21 *versus* 9 months	Combination with standard induction therapy	NCT00893373	Completed
			II	Patients age 60 or older with newly diagnosed FLT3/ITD‐mutated AML	Combination with standard induction therapy	NCT01253070	Active, not recruiting
			I/II	Patients with newly diagnosed AML irrespective of FLT3/ITD status receiving induction therapy in adults age 18–60 years	Combination with CLAG‐M induction	NCT02728050	Recruiting
			II	Patients age 60 or older with newly diagnosed AML who are ineligible for intensive therapy	Combination with azacitidine	NCT02196857	Recruiting
			I/II	Elderly patients with AML or high‐risk MDS	Combination with low‐dose cytarabine	NCT00516828	Completed, results not available
			I/II	Adult patients with newly diagnosed AML; 38% CR, 1‐year OS 74%	Combination with standard induction therapy	NCT00542971 32	Completed
		Quizartinib	II	Adult patients with relapsed/refractory AML with FLT3/ITD mutation	Single agent	NCT02984995	Recruiting
			III	Adult patients with relapsed/refractory AML with FLT3/ITD mutations *versus* salvage chemotherapy	Single agent	NCT02039726	Recruiting
			III	Newly diagnosed AML (adults age 18–75 years) with FLT‐ITD mutation receiving induction and consolidation chemotherapy, followed by maintenance	Combination with induction chemotherapy	NCT02668653	Recruiting
			I	Relapsed/refractory in adults age 18 or greater with AML irrespective of FLT3 status; 13% CR, 30% ORR	Single agent	NCT00462761	Completed
			I/II	Adult (age 18 or greater) patients with relapsed/refractory AML irrespective of FLT3 status	Combination with azacitidine or low‐dose cytarabine	NCT01892371	Recruiting
		Crenolanib	II	Relapsed/refractory AML (adults age 18 or greater) with activating FLT3 mutations	Single agent	NCT01657682	Recruiting
			II	Maintenance therapy after HSCT in FLT3‐positive AML in adults age 18 or greater	Single agent (maintenance)	NCT02400255	Recruiting
			III	Adult patients with relapsed/refractory AML with FLT3 mutations receiving salvage therapy	Combination	NCT02298166	Recruiting
			I/II	Adult patients with relapsed/refractory FLT3‐mutated AML receiving salvage therapy	Combination	NCT02400281	Recruiting
			II	Relapsed/refractory AML with FLT3 activating mutations in adults age 18 or greater	Single agent	NCT01522469	Completed, results not available
		Gilteritinib	III	Adult patients (age 18 or greater) with AML in CR1 following induction and consolidation	Single agent (maintenance)	NCT02927262	Recruiting
			III	FLT‐3‐mutated relapsed/refractory AML or CR with MRD in adults age 18 or greater	Single agent	NCT03070093	Available
			III	Maintenance therapy after allogeneic transplant in FLT‐ITD‐mutated AML in adults age 18 or greater	Single agent	NCT02752035	Not yet recruiting
			II/III	Azacitidine with or without gilteritinib in newly diagnosed AML age 18 or greater	Combination with azacitidine	NCT02997202	Recruiting
	RAS	Tipifarnib	II	Patients age 65 or older who are ineligible for intensive therapy	Single agent	NCT01361464	Completed, results not available
			I	Adult patients with relapsed/refractory AML or ineligible to receive intensive therapy	Single agent	NCT00101296	Completed, results not available
			II	Adult patients with poor‐risk AML who have achieved a CR after induction chemotherapy	Single agent (maintenance)	NCT00045396	Completed, results not available
			II	Adult patients 70 years or older with newly diagnosed AML who are ineligible for intensive therapy	Combination with etoposide	NCT00602771	Completed, results not available
			I/II	Adult patients with newly diagnosed AML	Combination with standard induction chemotherapy	NCT00096122	Completed, results not available
			II	Adult patients with relapsed/refractory AML	Single agent	NCT00354146	Completed, results not available
			II	Adult patients 70 years or older with newly diagnosed AML who are ineligible for intensive therapy	Single agent	NCT00093418	Completed, results not available
			II	Adult patients 60 years or older as post‐consolidation therapy	Single agent (maintenance)	NCT00048503	Completed, results not available
			III	Adult patients in second or greater remission OR patients greater than 60 years old in first remission; DFS 8.87 *versus* 5.26 months, OS 16.36 *versus* 9.27 months	Single agent (maintenance)	NCT00093470	Completed
		Selumetinib	II	Adult patients with relapsed/refractory AML	Single agent	NCT00588809	Completed, results not available
		Trametinib	II	Adult patients with relapsed/refractory AML or newly diagnosed AML who are ineligible to receive intensive therapy	Combination with Akt inhibitor GSK2141795	NCT01907815	Active, not recruiting
			I	Adult patients with relapsed/refractory AML or newly diagnosed AML who are ineligible to receive intensive therapy	Combination with AMG 232 or alone	NCT02016729	Active, not recruiting
		Rigosertib	I/II	Combination with azacitidine; dose escalation, dose expansion, safety	Combination with azacitidine	NCT01926587	Recruiting
	SYK	Entospletinib	Ib/II	Adult patients with newly diagnosed AML and relapsed/refractory disease	Combination with low‐ and high‐intensity regimens	NCT02343939	Recruiting
	Plks	Volasertib	III	Combination with low‐dose cytarabine in newly diagnosed AML age 65 and greater	Combination with low‐dose cytarabine	NCT01721876	Active, not recruiting
			I/IIa	Single agent and combination with low‐dose cytarabine in relapsed/refractory AML	Single agent and combination	NCT00804856	Active, not recruiting
Apoptotic targets	Bcl‐2	Venetoclax	III	Adult patients with newly diagnosed AML	Combination with azacitidine	NCT02993523	Recruiting
			III	Adult patients with newly diagnosed AML who are ineligible for intensive therapy	Combination with low‐dose cytarabine	NCT03069352	Not yet recruiting
			I/II	Patients 60 years and older with newly diagnosed AML who are ineligible for intensive therapy	Combination with low‐dose cytarabine	NCT02287233	Active, not recruiting
Stromal targets	CXCR4 and CXCL12	Plerixafor	I	Adult patients with newly diagnosed AML receiving induction chemotherapy	Combination with induction therapy (cytarabine and daunorubicin)	NCT00990054	Completed, results not available
			I	Patients 60 years and older with newly diagnosed AML	Combination with decitabine	NCT01352650	Active, not recruiting
			I	Adults patients with relapsed/refractory AML receiving salvage therapy; CR 46%	Combination with G‐CSF, mitoxantrone, etoposide, and cytarabine induction	NCT00906945 53	Completed
		Ulocuplumab	I/II	Combined with low‐dose cytarabine in newly diagnosed AML	Combination	NCT02305563	Active, not recruiting
			I	Safety and tolerability in patients with relapsed AML	Single agent	NCT01120457	Completed, results not available
Epigenetic	Hypomethylator	Guadecitabine	III	Adult patients with relapsed/refractory AML	Single agent *versus* treatment of choice	NCT02920008	Recruiting
	IDH1/2	AG‐221	III	AG‐221 *versus* conventional care regimens in patients 60 and older with relapsed/refractory AML and IDH2 mutation	Single agent	NCT02577406	Recruiting
			I/II	Adult patients with newly diagnosed AML with IDH1/2 mutations who are ineligible to receive intensive therapy	Combination with azacitidine	NCT02677922	Recruiting
			I	Adult patients with newly diagnosed AML receiving induction therapy with IDH1/2 mutation	Combination with induction and consolidation therapy	NCT02632708	Recruiting
	Bromodomain	OTX015/MK‐8628	I	Adult patients with AML or ALL with relapsed/refractory disease	Single agent	NCT01713582	Completed, results not available
		CPI‐0610	I	Adult patients with relapsed/refractory acute leukaemias	Single agent	NCT02158858	Recruiting
		FT‐1101	I	Adult patients with relapsed/refractory haematologic malignancies	Single agent	NCT02543879	Recruiting
	CDK	Alcvocidib	II	Alvocidib and cytarabine/mitoxantrone *versus* cytarabine/mitoxantrone in adults with relapsed/refractory AML with NOXA BH3 priming of ≥40% by mitochondrial profiling in bone marrow	Combination with induction therapy	NCT02520011	Recruiting
		Palbociclilb	I/II	Adult patients with MLL‐rearranged leukaemias	Single agent	NCT02310243	Recruiting

a Landmark trial that led to the approval of midostaurin for the treatment of FLT3 mutant AML by the U.S. Food and Drug Administration.

### KIT

Approximately 25% of core biding factor (CBF) AML patients carry gain‐of‐function mutations in the KIT gene. These mutations result in a constitutively active tyrosine kinase that contributes to aggressive leukaemia growth, and is associated with unfavourable outcome 23, 24. The German‐Austrian AML Study Group (AMLSG) and the CALGB 25 conducted phase II studies that evaluated dasatinib in combination with chemotherapy followed by 1‐year dasatinib maintenance in CBF AML. The CALGB 10801 study results suggest that outcome of KIT^mut^ patients approached those historically seen in KIT^wt^ patients, suggesting that dasatinib may overcome the negative prognostic effect of the KIT mutation. The AMLSG group is conducting a randomized phase III study adding dasatinib to induction chemotherapy in CBF AML. A French Intergroup study showed dasatinib used as single‐agent maintenance failed to prevent relapse in patients with poor molecular response or molecular recurrence following chemotherapy 26. The disappearance of KIT mutations at relapse suggests that clonal devolution may explain the absence of efficacy observed with single‐agent dasatinib.

### FLT3

The negative prognostic impact of the fms‐like tyrosine kinase receptor‐3 internal tandem duplication mutation (FLT3/ITD) on AML outcome and its physiologic effect of constitutive signalling through a receptor tyrosine kinase make it a highly desirable drug target. Mutational burden appears to predict addiction to FLT3 signalling and thus response to FLT3 inhibition 27. FLT3/ITD mutational burden is increased at disease progression rather than at presentation when the genomic composition of the AML is more heterogenous 28. In line with this finding, tumour cells derived from relapsed FLT3/ITD‐mutated AML patients appear to be addicted to signalling from the constitutively activated FLT3 receptor tyrosine kinase which insinuates that less specific inhibitors may be efficacious earlier in therapy, while more specific inhibitors may be best utilized at relapse 29. However, the optimal approach to incorporate FLT3 inhibitors into the management of newly diagnosed and relapsed/refractory FLT3‐mutated AML patients remains a matter of dispute and additional, pivotal studies are needed to provide an answer to this important question. *Midostaurin* is a multikinase inhibitor that claims the unique distinction of being the first FLT3 inhibitor proven to improve overall survival (OS) in FLT3/ITD‐mutated AML. As a single agent, Midostaurin treatment of 95 patients resulted in 1 partial and no complete remissions 30. However, when combined with conventional chemotherapy in newly diagnosed AML patients, midostaurin induced high remission and survival rates in both FLT3‐mutated and wild‐type patients 31. The CALGB conducted a randomized, placebo‐controlled Phase III trial (RATIFY) in treatment‐naive FLT3‐mutated AML patients <60 years encompassing induction chemotherapy and four consolidation cycles of high‐dose cytarabine combined with placebo or midostaurin, followed by midostaurin maintenance or placebo for 1 year 32. The median OS was 74.7 months for the group receiving midostaurin *versus* 26 months for the placebo group (*P* = 0.007). In addition, a 23% reduction in the risk of death was observed. The landmark results of this trial resulted in its FDA approval in combination with chemotherapy in AML patients younger than 60 years of age in April 2017. It is interesting to note that response rates to induction therapy did not differ significantly between treatment arms, suggesting prolonged exposure is required to benefit from the inhibitor. Moreover, patients randomized to midostaurin who underwent aSCT during the first remission had a survival curve plateau in the 60–70% range suggesting that aSCT remains a very relevant consideration in this population. Another interesting compound, s*orafenib,* was originally developed as an inhibitor of the serine/threonine kinase Raf but leukaemia clinical trials and physicians have capitalized on its off‐target inhibition of FLT3. Being FDA approved for hepatocellular carcinoma, it is the most widely accessible FLT3 inhibitor in clinical practice and frequently used off‐label. In younger patients, the addition of sorafenib to chemotherapy was well tolerated and showed preferential activity in FLT3‐mutated patients 33. The phase II randomized SORAML study in younger patients bore out these results 34 with improved EFS; however, grade 3–4 toxicities were higher in the sorafenib arm. The Study Alliance Leukemia trial combining induction chemotherapy with sorafenib in a randomized trial in patients over age 60 showed no difference in the event‐free survival (EFS) or OS between groups 35. This was attributed to higher induction mortality rate due to infectious complications in the sorafenib arm accompanied by lower protocol adherence for post‐remission therapy. These trials were FLT3 mutation agnostic and showed responses in FLT3‐wt patients supporting off‐target mechanisms of effect. In a Phase II study of relapsed or refractory FLT3/ITD‐mutated AML, the combination of sorafenib and the hypomethylating agent azacitidine yielded response rates of 46%, 36 suggesting that the combination of the two drugs may represent a clinically valuable regimen for relapsed, FLT3/ITD‐mutated AML in the elderly. The HMA backbone has the additional advantage of less up‐regulation of FLT3 ligand which is normally massively up‐regulated after cytotoxic chemotherapy and can compromise the efficacy of the FLT3 inhibitors. Sorafenib is being studied in the prevention of post‐transplant relapses, with an improved 2‐year progression‐free survival and a reduced risk of relapse 37 but data on timing and duration of therapy is sparse. Several other more specific FLT3 inhibitors are currently undergoing clinical studies. *Quizartinib*, an exquisitely specific FLT3 inhibitor, has a significantly longer half‐life than the above agents, as well as a greater capacity for inhibition of mutated FLT3 38. Several phase I and II studies have demonstrated encouraging activity of quizartinib in patients with relapsed/refractory AML 39, 40, 41. *Crenolanib*, a drug originally developed as an inhibitor of platelet‐derived growth factor receptor, has shown both activities in FLT3/ITD‐mutated AML and FLT3/ITD D835‐mutated AML 38. The D835 mutation has been identified as a potent mechanism of resistance to earlier FLT3 inhibitors. *Gilteritinib*, an agent with activity against wild‐type FLT3, FLT3/ITD, FLT‐TKD D835 and F691, as well as Axl, has been examined for the treatment of relapsed/refractory AML in two early‐phase clinical trials. In the phase I/II CHRYSALIS dose escalation trial, gilteritinib produced an overall response rate of 57% in FLT3‐mutated patients and 63% in patients with FLT3 mutations who received a dose of 80 mg per day or greater 42. In a follow‐up study of patients with relapsed/refractory AML, where 65% of subjects received >2 lines of therapy and 23% received treatment with a TKI, the overall response rate was 55% (60% for FLT3‐mutated patients and 29% for FLT3 wild‐type patients) in the setting of a median overall survival of 29 weeks 43. More recently, an exploratory analysis presented at the ASCO meeting in 2017 showed that molecular responses to gilteritinib in relapsed/refractory FLT3/ITD‐mutated patients correlated with the clinical outcome. In this study, Altman *et al*. 44 reported that patients with an ITD signal ratio of ≤10^−2^, 10^−3^ (major molecular response), or were MRD negative demonstrated a significantly longer median overall survival compared to patients who did not achieve a molecular response, suggesting that the ITD signal ratio may serve as a predictor of durable clinical benefit of gilteritinb.

### RAS

In AML, the RAS pathway is activated both by mutations occurring in *RAS* as well mutations and/or overexpression of upstream receptor tyrosine kinases such as *FLT3*. RAS inhibitors have had an underwhelming impact on AML. A phase 3 trial evaluating the farnesyltransferase inhibitor tipifarnib as first‐line therapy in older patients resulted in a CR rate of only 8%, and no survival benefit. A phase 2 trial of single‐agent selumetinib 45 showed modest activity only in the FLT3 wild‐type subset. The oral mitogen‐activated protein kinase kinase inhibitor trametinib showed more encouraging results with selective activity in NRAS or KRAS‐mutated AML and CMML 46. Response rates of 27% were seen in CMML and the lack of activity in RAS wild‐type leukaemias endorses the selective effect of the inhibitor. Rigosertib is a RAS‐mimetic interacting with the RAS‐binding domains of RAF kinases, preventing their binding to RAS and inhibiting the RAS‐RAF‐MEK pathway 47. This drug is being developed mainly in the MDS arena, and a phase III multicentre randomized trial is now comparing rigosertib to best supportive care in higher risk MDS progressing on HMA. Early results from a recent phase 1b study of the MDM2 inhibitor AMG 232 in 35 relapsed/refractory AML patients showed that AMG 232 was well tolerated and exhibited promising anti‐leukaemic activity (NCT02016729) 48.

### Polo‐like kinases

Polo‐like kinases (Plks) are involved in mitotic checkpoint regulation and cell division 49. Volasertib potently inhibits Plk1 as well as Plk2 and Plk3 blocking spindle formation and inducing cell cycle arrest in M phase. Volasertib was granted breakthrough therapy status by the FDA in 2013 for use with low‐dose cytarabine in high‐risk AML ineligible for standard therapy based on superior responses (31.0% *versus* 13.3%) in a randomized phase 2 study 50. However, the phase 3 POLO‐AML‐2 trial in the same population failed to meet the primary end‐point of superior responses 51 with an increased infection‐related mortality in the volasertib arm.

### Cyclin‐dependent kinase inhibitors

Alvocidib, a potent inhibitor of serine‐threonine Cyclin‐dependent kinases (CDKs) 9, 4 and 7, has been shown to be an active agent against AML 52. Pre‐clinical studies have demonstrated that inhibition of CDK9 and CDK7 leads to down‐regulation of transcripts of cyclin D1, c‐MYC and MCL‐1, leading to enhancement of anti‐tumour effects of cell cycle‐specific cytotoxic agents, such as cytarabine 53. Alvocidib has been studied in both the newly diagnosed and relapsed/refractory AML settings. To date, several clinical studies evaluating alvocidib in conjunction with cytarabine and mitoxantrone (FLAM) in patients with newly diagnosed AML have been published with overall CR rates of approximately 68% 54, 55, 56, 57, 58, 59. Of note, patients with favourable‐risk cytogenetic features such as core‐binding factor AML were excluded. In patients with relapsed/refractory AML, overall CR rates for FLAM were 36% 54, 55, 57, 60. Palbociclib, an inhibitor of both CDK 4 and 6, is currently being studied in leukaemia patients with MLL rearrangements. In a recently reported phase Ib study of six patients with relapsed/refractory leukaemia, one partial response, three disease stabilizations and two cases of the progressive disease were noted 61.

### Targeting apoptosis

Dysregulation of apoptosis in AML is partly mediated by overexpression of the anti‐apoptotic protein BCL‐2 and related family members. Venetoclax (ABT‐199) is a ‘BH3‐mimetic’ antagonist of BCL‐2. A phase 2 study of 32 patients with relapsed/refractory AML reported 5 CRs, the majority of which occured in patients carrying IDH1 or IDH2 mutations. The responses, however, were short‐lived 62. Improved responses in IDH‐mutated AML cases are attributed to 2‐hydroxyglutarate‐mediated inhibition of the activity of cytochrome oxidase in the mitochondrial electron transport chain, lowering the mitochondrial threshold to trigger apoptosis upon BCL‐2 inhibition 63. In a phase IB study in treatment‐naive older (≥65) patients with cytogenetically intermediate‐ or poor‐risk AML ineligible for intensive chemotherapy, the combination of venetoclax with HMA yielded an overall response rate of 76% 64. Venetoclax has been combined with low‐dose cytarabine in elderly AML producing high response rates (CR/CRi of 54%), with median survival not reached among the responders 65. This drug is garnering enthusiasm in the AML arena in combination with low‐intensity therapies in elderly patients.

### Targeting the stroma

Most of the progress in targeting AML–stroma interactions has been made by the development of CXCR4 inhibitors which mobilize leukaemic cells out of their protective niches by disrupting the AML–stroma interactions. These agents may also inhibit the pro‐survival signals provided to the blasts *via* CXCR4/CXCL12 signalling. In a phase 2 study, 46 patients treated with plerixafor in combination with chemotherapy showed a response rate of 46% (CR+CRi) associated with twofold mobilization in leukaemic blasts into the peripheral circulation 66. Ulocuplumab is a fully human IgG4 monoclonal antibody to CXCR4, with a half‐life longer than plerixafor well tolerated with salvage chemotherapy in relapsed AML 67.

### Epigenetics

Dysregulation of chromatin modifiers is a recurrent and sentinel event in oncogenesis. Strategies that target the recruitment and/or catalytic activity of these enzymes at chromatin represent an attractive therapeutic modality in leukaemia 68.

### DNMT inhibitors

The HMAs 5‐Azacytidine (azacitidine) and its deoxy analogue 5‐aza‐2′‐deoxycytidine (decitabine) are the two most extensively studied DNMT inhibitors and are approved for clinical use in haematologic malignancies in the United States. The cytidine nucleoside analogue Azacitidine which, upon cellular uptake, is in part converted into Decitabine, confers its cytotoxic effects via RNA and DNA incorporation, thereby disrupting protein and nucleic acid synthesis. DNMT inhibitors have been shown to induce response rates of 30% and more importantly prolong survival in elderly patients with AML in comparison with best available therapy for older patients 69, 70. Predicting responsiveness to this treatment modality has been challenging due to variable methylation profiles across biologic subgroups of AML. A recent phase 2 multicentre study showed that decitabine has preferential activity in p53‐mutated AML, one of the most chemotherapy resistant and unfavourable prognostic subsets of this disease. Moreover, detailed genomic analysis of the patients treated with decitabine showed robust suppression of the p53 mutant clone. These exciting data suggest an alternative up‐front strategy for the treatment of this group of high‐risk patients that will need to be verified in prospective trials. Guadecitabine, a dinucleotide of decitabine and deoxyguanosine and second‐generation hypomethylating agent, is currently under investigation for AML patients who are ineligible to receive intensive chemotherapy 71.

### IDH inhibitors

Neomorphic mutations in isocitrate dehydrogenase (*IDH1* and *IDH2),* each seen in 8–12% of AML cases result in an abnormal oncometabolite 2‐hydroxyglutarate, which leads to a hypermethylated genome with a resultant block in differentiation 72. The recently published phase 1/2 study of enasidenib (AG‐221), a first‐in‐class IDH2 inhibitor reported response rates of 40% and median duration of response of 4.8 months 73. This class of drugs induces differentiation of blasts rather than cytotoxicity and myeloablation. IDH differentiation syndrome was seen in 10% of patients and has also been reported with the IDH1 inhibitor ivosidenib (AG‐120) 74. While the drug potently suppresses the enzymatic activity of IDH2 and the levels of 2‐HG, it does not consistently suppress the allele burden of mutant IDH2. In fact, the emergence of mutant IDH2 neutrophils supports the idea of differentiation rather than elimination of the mutant clone. Enasidenib was equally effective in IDH2 R140 and R172 mutations. Certain mutational subsets of AML such as RAS mutations are more resistant to this therapy, and the role of mutational context in predicting response will continue to be explored. The IDHENTIFY phase III clinical trial is comparing enasidenib, to the standard of care for older patients with relapsed/refractory IDH2‐mutant AML. Both AG‐120 and enasidenib are also being investigated in newly diagnosed AML with *IDH1* and/or *IDH2* mutations, in combination with intensive chemotherapy, as well as with azacitidine in unfit patients. The 9.3‐month overall survival is also quite impressive in a pre‐treated population considering the expected 3‐month median survival in these patients 75. This class of drugs offers the exciting prospect of improving current standard of care in *IDH*‐mutant AML patients. Enasidenib has recently been approved by the FDA for the management of relapsed/refractory AML in patients with IDH2 mutations.

### HDAC inhibitors

Histone acetylase inhibitors work by altering chromatin structure and allowing transcription factors to bind to gene promoters. Romidepsin was one of the early HDAC inhibitors studied in a multicentre phase 2 study 76 in relapsed AML and was seen to preferentially induce differentiation in core‐binding factor AML cases. Vorinostat was more recently studied in combination with induction chemotherapy in a phase 3 trial, which was aborted due to lack of improvement over standard induction alone 77. However, it has been safely combined with azacitidine and has demonstrated efficacy in MLL‐rearranged AML at relapse with response rates of 35% 78 in this high‐risk subset of AML patients. Other oral HDACs, including entinostat and pracinostat, are in early trials in combination with HMAs. Of note, a recent study of entinostat combined with azacitidine showed pharmacodynamic antagonism, whereas prolonged administration of the hypomethylating agent alone appeared to increase response rates when compared to standard dosing 79.

### DOT1L inhibitors

Aberrant fusion proteins involving the MLL histone methyltransferase lead to recruitment of the histone methyltransferase DOT1L. Preclinical studies of DOT1L inhibition in MLL‐rearranged AML showed remarkable effectiveness; however, inhibition of DOT1L in a phase I trial with the small molecule Pinemetostat (EPZ‐5676) produced complete remissions in only 2 of 34 patients with an MLL‐rearranged leukaemia 80. Future studies of this agent might thus focus on combination regimens.

### Bromodomain inhibitors

The BET bromodomains are transcriptional coactivators involved in chromatin‐dependent signal transduction from master regulatory transcription factors to RNA polymerase II. The first direct‐acting bromodomain antagonist JQ1 was reported in 2010 81, and since then, the field has been expanding. BET recruitment is particularly relevant in MLL‐rearranged 82 and NPM1‐mutated AML based on proteomic studies. It has also shown synergy in combination with FLT3 inhibitors in preclinical testing in FLT3/ITD‐mutated AML 83. In a phase 1 study, the orally active BET inhibitor OTX015 was given to 41 elderly patients with relapsed/refractory acute leukaemia with five documented responses. Various other BET inhibitors have entered early clinical trials in patients with relapsed AML, including TEN‐010, GSK525762, FT‐1101 and CPI‐0610.

## AML heterogeneity and minimal residual disease

One of the major challenges to the sustained efficacy of targeted therapy is the genomic and cellular heterogeneity of AML. While bulk disease at initial diagnosis is comprised of a small number of dominant clones 84, this belies the underlying diversity of coexisting minor subclones that share some but not all of the gene mutations and epigenetic modifications present in the dominant clones 85, 86. Conventional cytotoxic chemotherapy or molecularly targeted agents can suppress or eradicate dominant clones leading to a complete remission but nevertheless facilitate the rise of genetically related but distinct clones either through selection of pre‐existing resistant subclones or clonal evolution and subsequent development of secondary resistance in otherwise sensitive clones leading to disease relapse 28, 87, 88. The frequency and stability at relapse of mutated genes that define the clonal architecture of AML are intimately related to its pathobiology. Pre‐leukaemic and leukaemic stem cells sequentially acquire mutations and diverge into subpopulations prior to frank transformation to AML 89, 90. Mutations in some genes, particularly those associated with epigenetic modification such as *DNMT3A* and *IDH2*, are acquired early in leukaemic development are therefore present in nearly all clonal progeny and are almost always retained in AML at relapse 91, 92. This contrasts with mutations in other genes such as *NRAS* and *FLT3* that are acquired late in AML pathogenesis and often lost at relapse 93, 94, 95, implying that residual pre‐leukaemic or leukaemic subclones that lacked those gene mutations rise to clonal dominance at relapse. This has significant implications for the development of targeted therapy as emergence of leukaemic clones that lack the targeted mutation may become a common resistance mechanism for inhibitors of the protein products of dispensable gene mutations acquired late in AML pathogenesis. In addition to genomic diversity, the cellular heterogeneity of AML complicates the development of targeted therapies. While the bulk of AML cells are morphologically and functionally defined as myeloid blasts, pre‐leukaemic and leukaemic stem and progenitor cells (LSPC) are both present during an overt clinical disease and persist in complete remission and are implicated as a source of relapse 90, 96. Targeted therapies which effectively kill AML blasts may not have activity against LSPC due to their increased quiescence and resistance to apoptosis. Furthermore, while therapies specifically directed at LSPC are in development, the immunophenotypes that clearly delineate them from normal hematopoietic stem cells are still uncertain and significant clonal diversity exists even within the LSPC compartment, suggesting that LSPC‐directed therapy may suffer from the same clonal escape that plagues treatment of bulk disease 97, 98. Given these challenges, preclinical testing with *in vitro* systems and *in vivo* xenograft models of AML has the potential to help guide the preclinical development of targeted agents that are effective in clinical trials as well as to understand mechanisms of therapy resistance. Recent improvements in the degree and scope of immunodeficiency as well as improved engraftment conditions have enabled more clinical specimens to be used in murine xenografts for preclinical testing 99, 100. However, despite these advances, some patient samples will fail to engraft; cells such as leukaemic blasts, progenitors and precursors that may be important in human disease cannot independently engraft in these mice which may overestimate the importance of leukaemic stem cells; and AML that does arise in these models is often restricted to a few clones that can obscure the clonal complexity or lack the most clinically relevant clones of AML in patients 100, 101. Another tool that may improve the development of targeted therapies is the emergence of high‐sensitivity methods of detecting minimal residual disease (MRD). Measurement of leukaemia‐associated aberrant immunophenotypes with multiparameter flow cytometry, gene fusion transcripts with quantitative polymerase chain reaction (qPCR) and gene mutations with qPCR, droplet digital PCR and next‐generation sequencing allows precise quantitation of as few as 1 in 100,000 residual aberrant hematopoietic cells in patients in complete remission depending on the platform used. MRD detection appears to offer robust prediction of relapse risk, particularly in the traditionally favourable core binding factor leukaemias and AML with *NPM1* mutations in the absence of *FLT3*/ITD mutations 102, 103, 104, 105, and is being further tested and validated in intermediate‐ and poor‐risk AML both in the setting of post‐induction remission assessment as well as prior to and following aSCT. Importantly, MRD measurement may be a powerful and underutilized tool for development of targeted therapies, especially in the resurgent concept of maintenance therapies during complete remission. Rather than rely on overt clinical relapse as the end‐point of induction and maintenance trials, tracking MRD longitudinally may provide a surrogate marker of response and allow detection of early molecular evidence of relapse or emergence of resistance mutations. In addition, many MRD monitoring methods are amenable for use with *in vitro* and *in vivo* treatment systems with the potential to inform the assessment of the efficacy of novel agents in preclinical models. The primary drawback to MRD testing, however, is the uncertainty of which clonal hematopoietic cells are being measured. These methods detect residual *disease* but also measure aberrant pre‐leukaemic and non‐leukaemic hematopoietic cells which have unclear biological and prognostic significance 106, 107. Further refinement of these methods will be critical to their usefulness both clinically and in pre‐clinical drug development.

## Conclusion

Although the tremendous progress in genetic technologies has brought more insight into the pathobiology of AML, there is still a knowledge gap with regard to the most suitable targets. The reasons for his knowledge gap are multifaceted and include the complex molecular architecture of the disease with multiple driver mutations and interconnected signal transduction pathways 108. Additional complexity is added by host‐specific factors such as the patient's age, comorbidities and psychosocial and socio‐economic status 109. However, biomarker adapted treatment protocols have already been established in several cancers but many therapies are only temporarily effective 110, 111, 112. Drug resistance to chemotherapy and targeted agents with subsequent relapse or progression thus remains a major problem in the treatment of cancer, including AML 113. Combination therapies offer the potential of targeting several pathways simultaneously to more effectively eliminate cancer cells and to prevent or delay the development of drug resistance. In appreciation of this concept, the ‘Beat AML Master Trial’, led by the Leukemia and Lymphoma Society in collaboration with several academic centres and the pharmaceutical industry, offers the hope to substantially boost the paradigm of personalized medicine in AML by utilizing companion biomarker‐based treatment strategies 114. In this trial, patients (n = 500 + ) with newly diagnosed AML will be assigned to targeted therapies after undergoing comprehensive genomic screening. Treatment arms consist of either the targeted agent alone or of the targeted agent combined with conventional therapy, such as standard ‘7 + 3’ or an HMA. Notably, patients whose AML cells lack a targetable lesion are eligible to receive novel therapy on a marker‐negative substudy. The ‘Beat AML Master Trial’ has enormous potential to further our understanding of the activity of currently available therapies in the treatment of AML. Despite this enthusiasm, however, it is noteworthy that, aside from expanding the boundaries of personalized medicine, the further development of already established FDA approved treatment protocols is critical to close our knowledge gap in optimizing the use of anti‐AML agents. This requires a global effort from physicians, scientists, insurance companies, pharmaceutical industry and regulatory authorities.

## Conflict of interest

The authors confirm that there are no conflicts of interest.
